# Transient Neurologic Symptoms following Spinal Anesthesia with Isobaric Mepivacaine: A Decade of Experience at Toronto Western Hospital

**DOI:** 10.1155/2018/1901426

**Published:** 2018-04-23

**Authors:** Ashwin Sankar, Minou Behboudi, Faraj W. Abdallah, Alan Macfarlane, Richard Brull

**Affiliations:** ^1^Department of Anesthesia, University of Toronto, Toronto, ON, Canada; ^2^Department of Anesthesia and Pain Management, Toronto Western Hospital, University Health Network, Toronto, ON, Canada; ^3^Department of Anesthesia and Keenan Research Centre, Li Ka Shing Knowledge Institute, St. Michael's Hospital, Toronto, ON, Canada; ^4^Department of Anaesthesia, Glasgow Royal Infirmary, Glasgow, UK; ^5^Department of Anaesthesia, Critical Care and Pain Medicine, University of Glasgow, Glasgow, UK

## Abstract

**Background:**

Transient neurologic symptoms (TNSs) can be distressing for patients and providers following uneventful spinal anesthesia. Spinal mepivacaine may be less commonly associated with TNS than lidocaine; however, reported rates of TNS with intrathecal mepivacaine vary considerably.

**Materials and Methods:**

We conducted a retrospective cohort study reviewing the internal medical records of surgical patients who underwent mepivacaine spinal anesthesia at Toronto Western Hospital over the last decade to determine the rate of TNS. We defined TNS as new onset back pain that radiated to the buttocks or legs bilaterally.

**Results:**

We found one documented occurrence of TNS among a total of 679 mepivacaine spinal anesthetics (0.14%; CI: 0.02–1.04%) that were performed in 654 patients.

**Conclusion:**

Our retrospective data suggest that the rate of TNS associated with mepivacaine spinal anesthesia is lower than that previously reported in the literature.

## 1. Introduction

Transient neurologic symptoms (TNSs), characterized by low back pain that radiates to the buttocks or legs after recovering from spinal anesthesia [1, 2], can be distressing to patients and providers. TNS typically appears within 24 hours of spinal anesthesia, lasts 2–5 days, and resolves completely without sequelae [2]. Because TNS is traditionally associated with intrathecal lidocaine, mepivacaine has been the local anesthetic of choice for short-acting spinal anesthesia at our institution for over a decade. While TNS following mepivacaine occurs less frequently compared to similar doses of lidocaine [3, 4], varied rates of TNS have been reported following mepivacaine spinal anesthesia. Previous small randomized trials examining patients undergoing isobaric intrathecal mepivacaine anesthesia for knee arthroscopy surgery have reported rates of TNS as high as 7.5% [3, 5–8]; however, intraoperative positioning for this procedure has been associated with an increased risk of TNS [1, 9]. It is our anecdotal experience that TNS following intrathecal mepivacaine is rare, occurring far less than the 6.4% incidence reported in one large prospective cohort of knee arthroscopy patients [9]. Therefore, we undertook this retrospective cohort study to understand the rate of TNS reported among surgical patients who underwent spinal anesthesia with isobaric mepivacaine at Toronto Western Hospital (TWH) over the last 10 years. We hypothesized that TNS following isobaric mepivacaine spinal anesthesia is rare.

## 2. Materials and Methods

Following the University Health Network ethics approval, we conducted a retrospective cohort study of consecutive patients who received spinal anesthesia with 1.5% or 2% isobaric mepivacaine for elective surgery at TWH between January 1, 2006 and March 31, 2017. We adhered to the STROBE recommendations for the design and reporting of observational studies [10].

We identified all mepivacaine spinal anesthetics by searching our regional anesthesia database, which captures data for all neuraxial blocks performed in the block procedure room at our institution. Patients who received multiple mepivacaine spinal anesthetics for more than one operative procedure in separate encounters during the study period and patients who received rescue spinal anesthesia using mepivacaine following failed spinal anesthesia with bupivacaine during the same encounter were both included.

All spinal anesthetics were performed by a regional anesthesia resident or fellow under direct supervision of the attending anesthesiologist or by the attending anesthesiologist. The dose and concentration of mepivacaine, along with any additives, were administered at the discretion of the attending anesthesiologist. Since 1.5% mepivacaine is not commercially available in Canada, this concentration was achieved by diluting 2% mepivacaine hydrochloride (Carbocaine® 2%, Hospira, Montreal, Canada) with preservative-free normal saline.

It is our institutional practice that any patient who develops neurologic symptoms following neuraxial anesthesia, including TNS, postdural puncture headache, and meningeal, neuropathic, or radicular symptoms, is referred to our anesthesiology department for immediate evaluation and management. Severe cases or patients at risk of acute deterioration are referred to the emergency department and/or for urgent neurosurgical consultation.

For each mepivacaine spinal anesthetic administered, the corresponding patient's internal medical record was reviewed for any documented indication of TNS postoperatively. We defined TNS as new onset back pain radiating to the buttocks or legs bilaterally [1, 2]. Specifically, we reviewed the initial surgical service postoperative follow-up note (hospital visit typically scheduled 2–6 weeks postoperatively); any postoperative anesthetic, medical, or surgical consultations; and any postoperative emergency room visit records. For patients who were unanticipatedly admitted to the hospital, the inpatient surgical service notes were reviewed for evidence of TNS. Patients for whom the medical record did not include any postoperative follow-up notes, consultation entries, or emergency records were excluded from the analyses.

### 2.1. Analysis

Descriptive statistics were generated for the entire sample using counts and frequencies for categorical variables and means, medians, standard deviations, and interquartile ranges for continuous variables. The distribution of continuous variables was assessed using the Shapiro–Wilk test of normality; none of the continuous variables were normally distributed, and medians with interquartile ranges are therefore reported. We describe the cases developing the outcome, and the absolute risk of developing the outcome with 95% confidence intervals is reported. In a secondary analysis, the characteristics of excluded patients were descriptively compared with those comprising the primary cohort. Statistical analyses were performed using SAS University Edition 9.4 (Cary, NC, USA).

## 3. Results

**Figure 1:**
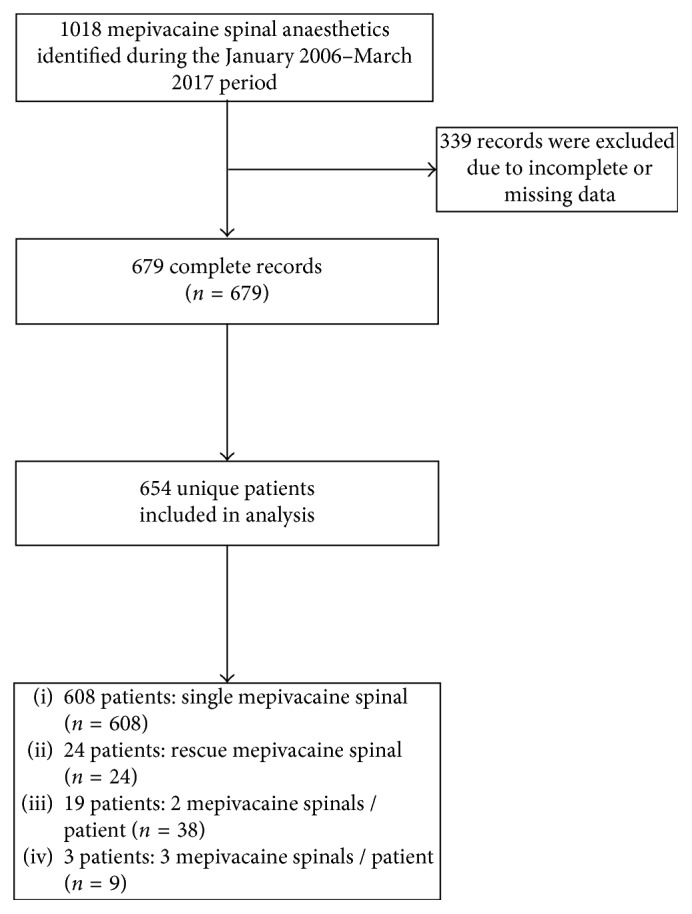
Flow diagram detailing the identification process for the present cohort of mepivacaine spinal anesthetics.

A total of 679 mepivacaine spinal anesthetics were performed in 654 patients. Twenty-two patients (3.3%) underwent multiple mepivacaine spinal anesthetics, among whom 19 (2.9%) received two mepivacaine spinal anesthetics and 3 (0.4%) received three mepivacaine spinal anesthetics, each in separate encounters. Twenty-four patients (3.6%) received a rescue spinal anesthetic using isobaric mepivacaine following a failed spinal anesthetic with isobaric bupivacaine (5–15 mg) during the same encounter.  Figure 1 details the identification process for the present cohort.

Our cohort characteristics are presented in Table 1. Among the 679 spinal anesthetics, 606 (89%) were performed using 2% mepivacaine and the remaining 73 (11%) were performed with 1.5% mepivacaine. 220 (32%) and six (0.9%) spinal anesthetics included intrathecal fentanyl (5–25 mcg) or morphine (50 mcg), respectively, in an admixture with isobaric mepivacaine. Four (0.6%) spinal anesthetics included hyperbaric bupivacaine (3.75–7.5 mg) in an admixture with isobaric mepivacaine for added baricity.

The spinal anesthesia procedures were most commonly performed in the sitting position (*n*=671, 99%), using a midline approach (*n*=660, 97%) with a 25 gauge (*n*=641, 94%) Whitacre needle (*n*=663, 98%). Paresthesia during intrathecal injection was reported during a single spinal anesthetic, while pain upon intrathecal injection was not reported during any spinal anesthetic. No patients experienced any new postoperative motor deficits.

The median time to the initial postoperative surgical visit was 20 days (IQR 13–47 days). One patient was referred to our anesthesia department for consultation regarding postoperative neurological symptoms. None of the remaining 653 patients (who underwent 677 mepivacaine spinal anesthetics in total) received postoperative consultation by any medical or surgical service at our institution, none were referred to our emergency room, and none were readmitted postoperatively.

### 3.1. Primary Outcome: TNS

Among the 679 mepivacaine spinal anesthetics performed, a single occurrence of TNS (0.14%; CI: 0.02–1.04%) was found in a 75-year-old female patient (BMI 27) undergoing ankle hardware removal surgery, who received spinal anesthesia with 2.5 mL of 2% mepivacaine at the L4-L5 level. No pain or paresthesia was reported during intrathecal injection. Of note, this patient appeared in our dataset twice, as she received a mepivacaine spinal anesthetic in a separate encounter for knee arthroscopy 43 months prior with no complications, but developed TNS following second exposure.

In secondary analyses (Table 2), the characteristics of excluded patients did not qualitatively differ from the primary study cohort.

## 4. Discussion

Our single-institution retrospective cohort study suggests that the reported rate of TNS following spinal anesthesia with isobaric mepivacaine is very low. Our large cohort of mepivacaine spinal anesthetics allowed for addressing some important shortcomings in previous small randomized trials, wherein a wide range of 0–7.5% rates of TNS have been observed [3, 5–8]. The majority of previously published studies were small, each including less than 100 patients [3, 5–8]; and several of these studies employed a combined spinal-epidural technique which may have obscured the relationship between spinal mepivacaine and TNS [6–9]. Further, almost all previous studies investigating the association between spinal mepivacaine and TNS were conducted in patients undergoing arthroscopic knee surgery [3, 5–9], the intraoperative surgical position for which has been associated with an increased risk of TNS [9]. In our study, 38% of mepivacaine spinal anesthetics were performed for knee arthroscopy procedures; the remaining spinal anesthetics were performed for lower extremity orthopedic and lower abdominal procedures. This variety of surgical procedures may have contributed to the low rate of TNS observed herein. Our low rate of TNS may also have been impacted by contemporary anesthetic practice, including the adoption of multimodal analgesia and prophylactic antiemetic medications as evidence supporting their use became available. It is possible that the increasingly routine use of nonsteroidal antiinflammatory drugs such as ketorolac administered for preventive analgesia and the inherent antiinflammatory properties of dexamethasone administered for postoperative nausea and vomiting may prevent and/or mask symptoms of TNS, and at least partially account for our low reported rates of TNS. The latter notwithstanding, the results of this retrospective review underscore that TNS following mepivacaine spinal anesthesia has not posed a clinical challenge to our regional anesthesia teaching program over the last decade.

Under the conditions for detection of TNS in the present study, a single case was identified. The single observed case of TNS occurred in an elderly patient who received mepivacaine spinal anesthesia for ankle hardware removal after 43 months of having received mepivacaine spinal anesthesia for knee arthroscopy. While advanced age is independently associated with TNS [9], the effect of multiple exposures to intrathecal mepivacaine or rescue mepivacaine spinal anesthesia has not been extensively investigated. Unfortunately, although our cohort captured the greatest number of patients receiving multiple isobaric mepivacaine spinal anesthetics to date, the single observed occurrence of TNS precluded examining statistical associations of previously identified risk factors for TNS such as age [9] or novel considerations such as multiple exposures.

Our results are subject to several limitations. First, our study comprised all mepivacaine spinal anesthetics conducted in a single-academic tertiary care hospital in a manner reflective of institutional clinical practice. Similar studies at centres with differing case mixes are necessary to generalize our findings to other practice settings. Second, due to the retrospective nature of our study, there is considerable potential for underreporting. It is possible that minor episodes resolved spontaneously and may not have been recalled, reported, and/or documented. Given that TNS is routinely discussed and disclosed during our informed consent process prior to performing spinal anesthesia using mepivacaine, it is also possible that even moderate-or-severe TNS symptoms were appropriately disregarded and unreported by patients. Further, we excluded 339 mepivacaine spinal anesthetics from our analyses due to incomplete or missing postoperative data, most likely because follow-up occurred in a private clinic. However, none of these patients were referred to our anesthesia department for consultation, none received any other medical or surgical consultation, none were readmitted, and none visited our emergency room postoperatively. It is also noteworthy that the characteristics of excluded patients (Table 2) did not appreciably differ from the present study cohort. In addition, though our dataset includes patients undergoing several surgical procedures, given the single case of TNS observed, we are unable to statistically verify known risk factors for TNS, such as lithotomy position and obesity [1].

## 5. Conclusion

A large cohort comprising a decade of experience with isobaric mepivacaine for short-acting spinal anesthesia at our institution suggests that the rate of TNS is lower than that previously reported in the literature. Practically, TNS following mepivacaine spinal anesthesia has not posed a clinical challenge to our regional anesthesia teaching program over the last decade.

## Figures and Tables

**Table 1:** Cohort characteristics (*n*=679 mepivacaine spinal anesthetics).

Age (in years; median, IQR)	61	71
Sex (*n* male, %)	336	49%
Height (cm; median, IQR)	168	161–177
Weight (kg; median, IQR)	86	71–100
BMI (kg/m^2^; median, IQR)	29	25–35
Mepivacaine volume (mL; *n*, %)		
≤1.5	81	13%
1.6–2.0	64	9%
2.1–2.5	178	26%
2.6–3.0	336	49%
>3	20	3%
Spinal position (*n*, %)		
Sitting	670	99%
Lateral	9	1%
Spinal approach (*n*, %)		
Midline	660	97%
Paramedian	19	3%
Spinal level (*n*, %)		
L2-L3	58	8%
L3-L4	466	69%
L4-L5	153	23%
Not defined	2	0%
Spinal needle type (*n*, %)		
Quincke	6	1%
Sprotte	10	1%
Whitacre	663	98%
Spinal needle Gauge (*n*, %)		
<24	26	4%
25	641	94%
27	11	2%
Not defined	1	0%
Surgical procedure (*n*, %)		
*General surgery*		
Inguinal hernia repair	4	1%
*Orthopedics*		
Ankle		
Arthroscopy	29	4%
Fusion	2	0%
Hardware removal	56	8%
ORIF	45	7%
Tendon repair	24	4%
Foot		
1st MTP surgery	36	5%
Reconstruction	60	9%
Hip		
Arthroplasty	4	0%
Arthroscopy	6	1%
Knee		
Arthroplasty	68	10%
Arthroscopy		
Bilateral	31	5%
Unilateral	226	33%
Hardware removal	39	6%
ORIF	13	2%
*Plastic surgery*		
Soft tissue surgery^∗^	10	1%
*Urology*		
Cystoscopy or cystolithopaxy	12	2%
TURP or TURBT	14	2%
Intraoperative position (*n*, %)		
Knee arthroscopy	257	38%
Lateral	17	2%
Lithotomy	27	4%
Prone	7	1%
Supine	371	55%

BMI = body mass index; MTP = metatarsal-phalangeal joint; ORIF = open reduction internal fixation; TURBT = transurethral resection of bladder tumor; TURP = transurethral resection of the prostate; ^∗^carcinoma excision, hematoma evacuation, and skin graft excision.

**Table 2:** Characteristics of excluded records (*n*=339 mepivacaine spinal anesthetics).

Age (in years; median, IQR)	54	44–63
Sex (*n* male, %)	190	56%
Height (cm; median, IQR)	171	164–179
Weight (kg; median, IQR)	93	74–110
BMI (kg/m^2^; median, IQR)	31	25–38
Mepivacaine volume (mL; *n*, %)		
≤1.5	22	7%
1.6–2.0	17	5%
2.1–2.5	82	24%
2.6–3.0	213	63%
>3	5	1%
Spinal position (*n*, %)		
Sitting	338	100%
Lateral	1	0%
Spinal approach (*n*, %)		
Midline	331	98%
Paramedian	8	2%
Spinal level (*n*, %)		
L2-L3	25	7%
L3-L4	232	68%
L4-L5	79	24%
Not defined	3	1%
Spinal needle type (*n*, %)		
Quincke	2	0%
Sprotte	6	2%
Whitacre	331	98%
Spinal needle Gauge (*n*, %)		
<24	13	4%
25	323	95%
27	1	0%
Not defined	2	1%
Surgical procedure (*n*, %)		
*General Surgery*		
Inguinal hernia repair	3	1%
*Orthopedics*		
Ankle		
Arthroscopy	40	12%
Fusion	7	2%
Hardware removal	26	8%
ORIF	36	11%
Tendon repair	11	3%
Foot		
1st MTP surgery	6	2%
Reconstruction	10	3%
Hip		
Arthroplasty	0	0%
Arthroscopy	4	1%
Knee		
Arthroplasty	33	10%
Arthroscopy		
Bilateral	7	2%
Unilateral	122	36%
Hardware removal	11	3%
ORIF	10	3%
*Plastic Surgery*		
Soft tissue surgery^∗^	5	2%
*Urology*		
Cystoscopy or cystolithopaxy	2	0%
TURP or TURBT	6	2%
Intraoperative position (*n*, %)		
Knee arthroscopy	129	38%
Lateral	8	2%
Lithotomy	7	2%
Prone	3	1%
Supine	192	57%

BMI = body mass index; MTP = metatarsal-phalangeal joint; ORIF = open reduction internal fixation; TURBT = transurethral resection of bladder tumor; TURP = transurethral resection of the prostate; ^∗^carcinoma excision, hematoma evacuation, and skin graft excision.
